# Annual Meeting of the British Medical Association, at Glasgow

**Published:** 1888-09

**Authors:** Wm. B. Meany

**Affiliations:** Glasgow


### THE BRITISH MEDICAL ASSOCIATION.

The British Medical Association assembled August 7 in the University Buildings in the city of Glasgow, Scotland.

The first day of the fifty-sixth annual meeting was chiefly devoted to meeting of councils, reading of reports of various committees, etc.

The chair was occupied by the retiring President, Dr. J. T. Banks.

In the evening, Professor W. T. Gairdner, President-elect, delivered the annual address on "The Physician as a Naturalist." He closed his address with one of those authoritative utterances respecting the relations of science and religion which in these days of confusion and spiritual trouble are listened to with much avidity and eager hopefulness. President Gairdner said: "Probably there may be some of you present who have been led to take note of a proverb which I am bound to say I have not been able to trace to its source, but which I suspect to have been the growth of a more mediæval period. '*Ubi tres medici, duo athei.*' Irreverence in a physician, he maintains, disqualifies him for some of his noblest functions. Indifference makes him an anomaly in our social system. It will be in the future his high privilege to play the part of a mediator between destructive and constructive influences of the times. Remembering the pernicious despotism exercised over his own art, he will study the Bible emancipated from fetters of tradition. His relations with suffering and the solemn ministration of life and death will keep him in a region 'in which from day to day the voices of the unseen world (if he will only listen) will be ever sounding close to his ears.' And so in the highest sense a spiritualist, working according to the strict method of the materialist, he may in the evolution of more certain and far-reaching art, master the relations between our physcial and moral natures, and whilst healing fleshly ills acquire the skill to treat by medicine the many varieties of moral *disease* as systematically as he now treats physical ills."

Promptly at 10.30 a. m. on Wednesday, the various sections—twelve in number—met, remaining in session daily until 2 p. m. during the week.

SECTION A—MEDICINE.

Professor T. McCall Anderson, M. D., presided and delivered the opening address on "The Diagnosis and Treatment of Syphilitic Affections of the Nervous System." After quoting certain views regarding the affection, he said his view of syphilis was that, as "mild" syphilis was apt to be treated in a cavalier fashion and without due care and persistence, disease of the nervous system was apt to follow in its wake; and that such disease was much more common than was generally supposed. He then proceeded to deal with his subject under the following headings: The entrance of the syphilitic poison into the system; the discovery of other manifestations of the disease and traces of by-gone syphilitic manifestations; the feeling of pain and appearance of patient; the age and sex of the patient, as being important; the occupation of the patient; the fact that syphilitic nervous lesions are frequently multiple; the variability of the symptoms; the fundamental types recognized as cerebral in syphilis; eye-symptoms, and the general treatment.

A discussion followed.

Dr. Andrew Smart, of Edinburgh, opened the section of medicine on "Some Undescribed Forms of Respiratory Neurosis." The function of respiration undergoes remarkable changes when the nerves which regulate it become disordered from any cause, or when their normal activity is in any way interfered with. The first case referred to was that of spasm brought on by slight emotional disturbances. The spasm lasted fifty seconds, and in that space the patient breathed 132 times, while during the same interval the heart registered 212 pulsations. This case was only one of a group which Dr. Smart cited to illustrate the effects resulting from vaso-moter spasm of a particular seat of the brain. Referring next to a second class, it was explained that the vagus and phrenic nerves (the two chief nerves of breathing), from their combined control of the entire respiratory movements, were a prolific source of many and sometimes grave disorders, not only of breathing, but of the respiratory muscles, implicating sometimes all the other muscles in general convulsive spasm, and suspension of breathing for a time. This mode of disturbance he described as "Inspiratory Neurosis," inasmuch as when these nerves were so excited respiration was always stopped in the position of full inspiration. The third disorder he referred to was that of nerve expiration. Irritations of this nerve or its centre excited persistent cough, going on until spasm and threatened suffocation was imminent. This condition as a symptom was characteristic of a number of maladies, especially whooping-cough, which he said might be viewed as a disordered state of that nerve. A fourth respiratory neurosis was described under the designation of "Respiratory Dysphagia" connected with the functions of swallowing. It was explained that it had been definitely ascertained that there was a nerve whose function it was to stop breathing during the act of swallowing, and interference with that action caused difficulty of breathing during meals. The troubles were of different kinds, which were fully described according to the nature of the disorder of the nerve. He concluded by referring to "Cerebral" and Cheyne-Stokes breathing, the only two modes of abnormal respiration. He showed by means of respiratory tracings that the former were misconceived and wrongly interpreted as a sign of disease, and that it was generally confused with that of Cheyne-Stokes respiration. The latter, he remarked, was characterized by rythmical periodicity, a difference of great significance absent in the other, which is continuous respiration. He considered the occurrence of Cheyne-Stokes breathing of the true periodic type as a sign of the greatest import. He furthermore believes that the stoppage of periodic character of the breathing is brought about by stimulation of a distinct centre of respiratory inhibition.

A large number of papers were read in this section.

SECTION B—SURGERY.

Professor George Buchanan, M. D., presided. In the course of the opening address he stated that he was the first person who ever adopted the practice of surgery to the exclusion of all the other branches of medicine. In this he was joined by Sir Joseph Lister and Sir G. H. B. Macleod; so that the practice of surgery was now adopted by others who had obtained hospital appointments—he thought to the advantage of the profession and the medical school. No one, he said, who had had so long experience as himself could fail to be struck with the rapid strides with which the science and practice of surgery had advanced. His early years were associated with administration of anæsthetics, for he was the first person in Scotland who was made insensible to pain by the administration of ether—an experiment he performed upon himself. In speaking of the tendency of surgery within the last thirty years, he said he thought that period more than any previous one had been characterized by scientific research and had profited by the investigations of pathological histology. It might seem a little late in the day to refer to antiseptic surgery as one of the advances of the present time, but it was not out of place to do so in this University, where the principle was first enunciated, nor at this meeting, where Sir Joseph Lister was to be present. It was while he held the chair of surgery here, from 1860 to 1869, that he made those observations by which the germ-theory of putrefaction was established and which culminated in the antiseptic treatment of wounds. The discovery in the later part of the period which perhaps had caused most interest was that of bacillus of tubercle, not only because it showed the germ-origin of a disease long believed to be constitutional, but because it betokened the probably similar origin of many allied affections, such as syphilis, cancer, etc. Of course a bacillus can only live in a suitable pabulum supplied by a peculiar constitution, so that it was to be considered only as an exciting cause acting in a predisposed part. In pathological histology the researches into local beginnings of cancer had greatly cleared the way for a more precise etiology than formerly; still it must be admitted that that dread disease still baffled the most skillful, both as to its origin and progress. Operations upon the head, chest and abdomen were now daily practiced which twenty years ago would have been deemed unjustifiable. Might he take the liberty of saying that in some of these the limit of prudence had been reached, if not exceeded? Especially in cases of malignant disease, where it could be recognized, the utmost caution was required in determining for or against the operation.

A number of papers of professional interest were read in this section.

One of the most important papers read at the meeting was that of Dr. William MacEwen, of Glasgow, "On Surgery of the Brain and Spinal Cord." As introductory to his lecture he briefly sketched the history of the evolution of cerebral surgery. Lesions of the head had at all times held a prominent place in the annals of surgery. Much had been done by surgeons before the present day to advance the healing art as applied to this particular region, but their efforts were chiefly directed to the superficial parts, and their operations were simple and undertaken for the most part upon the primitive evidence of direct visual and tactile observations. No references were made by them to cerebral surgery as now known. There were two formidable barriers to the advance in this region; first, the fact that the majority of intra-cranial operations were attended by inflammatory action, which so often proved fatal as to cause the surgeon to shun active interference; and secondly, the brain was a dark continent in which they could descry neither path nor guide capable of leading them to a particular diseased area, and if they did attempt to reach it, it could only be by groping in the dark. The cerebrum was supposed to perform its functions as a whole in the same way as the liver and kidneys performed theirs, there being no differential of function. In recent years abundant proof had been gathered from human pathology to put beyond serious doubt the broad fact that there are points in the human cortex cerebri intimately related to the motor and sensory functions of certain parts of the body. The apportionment of definite parts and their precise delimitation was still the subject of investigation. For the purposes of the paper, it was enough to recognize that there were certain regions of the brain in intimate relation with the movements and sensations of certain parts of the body, and which in the presence of either irritative or destructive lesions gave rise to phenomena which were of the greatest diagnostic value. This extended physiological knowledge enabled cerebral lesions to be more accurately localized, whilst his experience showed by preserving aseptic the parts operated upon surgical interference with the brain could be robbed of its chief dangers. In tracing the progress of cerebral surgery, he cited a number of interesting cases which had come under his own observation, chiefly of tumor and abscess of the brain. Dealing next with the present aspect of cerebral surgery, he inquired if the localising motor phenomena were reliable guides to diagnosis of cerebral lesions situated in the motor cortex. His answer was an unhesitating affirmative. Each case, however, required to be studied on its own merits; the whole phenomena presented; the unobstrusive as well as the prominent features must be carefully searched for; the degree in which each was present must be accurately measured; and the whole weighed and compared with former experience before drawing a conclusion. From his own experience as well as cases reported by Mr. Godlee, Mr. Horsley, and many others, it was also clear that the motor and sensory phenomena formed reliable guides to the localization of lesions in cerebral convolutions. He adduced a number of instances, one of special interest, showing that the diagnosis of cerebral lesions in non-motor regions might be made from sensory phenomena; and also as an example of the difficulty of finding the exact cue to the lesion, and how easily it might be overlooked. He said a man who had received an injury about a year previously suffered from deep melancholy, strong homicidal impulses, relieved by paroxysms of pain in the head of indefinite seat. Though the pain was excruciating, he welcomed it, as it temporarily dispelled the almost irresistible desire to kill his wife and children and other people. Prior to receiving this injury he was perfectly free from impulses of this kind, and had led a happy life with his family. Behind the angular process of the frontal there was a slight osseous depression, which could not account for his symptoms. There were no motor phenomena, but on minute inquiry it was discovered that immediately after the accident, and for almost two weeks subsequently, he suffered from psychical blindness. Physically he could see, but what he saw conveyed no impression to his mind. These phenomena, however, gave the key to the hidden lesion in his brain. On operation, the angular gyrus was exposed, and it was found that a portion of the internal table of the skull had been detached from the outer, and had exercised pressure on the posterior portion of the supramarginal convolution, while a corner of it had penetrated and lay embedded in the arterior portion of the angular gyrus. The bone was removed from the brain, and re-implanted in the proper position, after which he became greatly relieved in his mental state. Though still excitable, he had made no further allusions to his homicidal tendencies, which previously were obtrusive, and he was now at work. Such cases of complete mind-blindness were rare, and the definite localization in this case would assist in indicating in a man what the arterior portion of the angular gyrus, and the posterior portion of the supramarginal convolution, subserve. In cerebral surgery not only did one require to localize the lesion, to select suitable cases, but also, after exposing the brain and its lesions, to judge when to advance and when to hold the hand. After dealing with such topics as the possibility of the removal of large pieces of the motor area, anchoring of the brain, false hernia cerebri, and re-implantation of bone to fill the hiatus in the skull left by injury or made by operation, he turned to another portion of the nervous system. It was found that certain sensory and motor phenomena, due to lesions in the spinal cord, were amenable to operations, and were attended by a measure of success sufficient to offer a prospect of relief to a distressing class of sufferers hitherto regarded as hopeless. The spinal membranes and the cord itself could be exposed, and neoplasms and encroachments upon the lumen of the canal might be removed therefrom without unduly hazarding life. Such interference was unsparingly condemned by writers on the subject, their remarks, however, being applied to injuries, as no such operation had been hitherto contemplated upon idiopathic cases. They contended that they were full of danger, being difficult, prolonged, and attended with profuse hæmorrhage; secondly, that the operation could hardly benefit the patient; and thirdly, that no one had been able to present a successful case. Each of these points had now lost its validity. He gave instances of successful operations on the spine, and, in in conclusion said, that the same phenomena by which they were now able to recognize certain cerebral lesions, and locate them in precise areas, were exhibited by patients who came under the eye of their surgical predecessors, some of whom must have the album of memory filled with such impressions. Yet they saw not their import. They were so hampered by circulated physiological dogma of the time that their true significance never dawned upon them. The facts were reflected from their brains as images from a mirror, and no more.

STATISTICAL RÉSUMÉ.

Of twenty-one cerebral cases, exclusive of fractures of the skull or other immediate effect of injury, operated upon by Dr. Macewen, there had been three deaths and eighteen recoveries. Of those who died, all were *in extremis* when operated on. Of the eighteen who recovered, sixteen are still alive in good health and most are at work, leaving two who have since died—one eight years after operation of Bright's disease, but having in the interval been quite well and able to work; the other forty-seven days after the operation, after the abscess was perfectly healed, from an acute attack of tubercular enteritis.

SECTION C—OBSTETRIC MEDICINE.

Thomas More Madden presided, and read a valuable address, "The Rise and Progress in Obstetric Medicine." A number of papers were read in this section, among which might be mentioned one "On Intrauterine Death; Its Pathology and Preventive Treatment." An active discussion ensued on all papers read in this section; communications bristling with technicalities were made by nearly all members present.

SECTION D—PUBLIC MEDICINE.

Henry Duncan Littlejohn, M. D., President. He discussed "Sanitary Legislation" by reading a somewhat lengthy paper of great importance and interest.

SECTION E—PSYCHOLOGY.

James C. Howden, M. D., presided. In his opening address he first treated of the lesions derived from the few cases of insanity in which surgical interference had resulted in recovery, and then proceeded to discuss the mental and medical treatment of insanity.

SECTION F—ANATOMY AND PHYSIOLOGY.

John Cleland, M. D., presided and delivered an interesting address which was followed by general discussion.

SECTION G—PATHOLOGY.

Sir William Aitken, M. D., presided. He read a valuable paper on "The Progress of Pathology." In closing, he remarked that clinical specificity in disease had been shown to be only partially true. Professor Cruikshank read a paper on "(1) Anthrax in swine; (2) Tuberculosis in swine; (3) Human and Bovine Tuberculosis."

SECTION H—OPHTHALMOLOGY.

Thomas Reid, M. D., presided, and Dr. R. Brudenell Carter opened a special discussion on the "Treatment of Senile Cataract." The following gentlemen took part in the discussion: Messrs. Power, Carter, McHardy, Teale, Lloyd-Owen, Hewetson, Grossman, Bell, Snell, Wolfe, and others. It was concurred in by all members present, that all operations should be done under full antisepsis. Extraction of cataract with iridectomy, or a preliminary iridectomy, was insisted upon by a large number of members, whilst the extraction of cataract without iridectomy appeared to have many advocates. Mr. Powers suggested that if nature designed other than a round pupil for visual purposes she would have made it otherwise; he thought the pupil left by an iridectomy (key-hole shape) was contrary to nature for visual purposes. That the artificial pupil was in his mind no doubt the cause of a large number of astigmatic cases met with after iridectomy. A wide iridectomy should be made, permitting easy escape of the lens and lens débris and less risk of bruising the iris by pressure of lens in its escape through an opening made by incision. Graefe's knife with Graefe's modified linear incision was accepted as the least dangerous operation. The following are some of the papers read in this section: "A Contribution to the Study of Hemianopsia of Central Origin, with Special Reference to Acquired Color Blindness"; "On General Neuroses. Having an Ophthalmic Origin"; "On (1) Sailors and their Eyesight; (2) Unusual Corneal Opacity in Process of Recovery"; "On Color-Blindness"; "On the Value of Cautery in Treatment of Ulcerations of Cornea"; "Three Cases of Conical Cornea treated by Actual Cautery"; "On Cocaine," etc. Mr. R. Brudenal Carter described his operation for "Opening the Sheath of the Optic Nerve for Relief of Pressure," and exhibited a case of instruments specially devised for his operation, which is as follows: Tenotomy of the recti and a hook introduced through the opening so as to embrace the optic nerve. The eyeball is rotated so as to expose the optic nerve; an incision is made in sheath of the nerve, evacuating the fluid; a small probe-pointed bistoury is passed into the opening and the sheath of the optic nerve is slit up along its entire length; the divided rectus muscle is brought in apposition as well as the conjunctival wound by silk sutures. Antiseptic precaution is observed. The eye is then bandaged and the patient is to remain quiet for a week. He says this operation is harmless, and that the relief from pain experienced by relieving tension can not be overestimated.

SECTION I—OTOLOGY.

Thomas Barr, M. D., presiding. The principal discussion in this section was that on "Adenoid Growths in the Naso-pharynx; their Influence on the Middle Ear and their Treatment." These growths it was thought best to remove by the finger-nail and also using an artificial finger-nail when required. Hartman's curette and forceps were sometimes required in the removal of these growths. Anæsthetics in removal of growths from naso-pharyngeal or buccal walls of pharynx or in any surgical interference where hæmorrhage was likely to ensue, should not be used, though in some cases, especially in children, it was considered impossible to operate without them. Two cases of death were reported from hæmorrhage into trachea in removing these growths, the patients in both instances being under the influence of chloroform. For relief of pain reliance should be placed upon the free use of a 4 to 10 per centum solution of cocaine.

SECTION J—DISEASES OF CHILDREN.

Walter Butler Cheadle, M. D., presided. He said he did not advocate the establishment of children's diseases as a distinct specialty. This section was amongst the most prominent and was largely attended. Professor A. Jacobi, of New York, started a discussion on diphtheria and was followed by Professor Gairdner, Professor Buchanan, and many others. Professor F. E. Waxham, of Chicago, explained the proper method of intubation of the larynx. Members from many of the sections came specially to hear, and probably for the first time heard a description of the proper intubation of the larnyx, etc.

SECTION K—PHARMACOLOGY AND THERAPEUTICS.

James Morton, M. D., presided. There was discussion on carbolic acid, antipyrin, antifebrin, and their allies, especially as regards their antipyretic, analgesic, and antiseptic actions. Papers were read on Phenacetin, coca, alkaloids, etc.

SECTION L — LARYNGOLOGY AND RHINOLOGY.

Felix Semon, M. D., presided. A discussion on "The Causes, Effects, and Treatment of Nasal Stenosis" was opened by Dr. McIntyre, of Glasgow. Papers were read on "The Pathology of Echondrosis of the Triangular Cartilage with a New Operation"; Removal of Nasal Polypi"; "Hay Fever," etc.

REPORT OF SCIENTIFIC GRANTS COMMITTEE.

The following are reports made to the committee:

Dr. C. E. Beevor and Mr. Victor Horsley have been carrying on investigations of an experimental character in stimulating the cortex and the internal capsule, as well as investigations on the brachial plexus of the monkey. These observations are not furnished for publication, as further work on the subject is required. They have also finished an investigation on some of the functions of certain cranial nerves (v, vii, ix, x, xi, xii), and of the first three cervical nerves in the monkey, which has been read before the Royal Society. The chief points in this paper are that the soft palate does not receive its motor supply from the seventh nerve, but from the accessory nerve of the vagus; the hypoglossal nerve supplies only one-half of the tongue, and it does not supply the depressors of the hyoid bone, through the descending noni branch. The depressors of the hyoid bone are supplied by the first and second cervical nerves, of which the branch from the first cervical to the hypoglossal supplies, especially the sterno-hyoid, and sterno-thyroid, while the omo-hyoid gets its nerve supply especially from the branch from the second cervical nerve to the descendens noni. The nerves were stimulated by the Faradic current in the cranial and spinal cavities, and also in the neck.

Dr. Hadden and Mr. Ballance, by their stimulating experiments, have led to conclusions of a positive and negative kind. They have found that stimulation of the prefrontal region, as well as the temporo-sphenoidal and occipital lobes, has not been followed by movements. They are inclined to think, also, that the ascending parietal convolution and the angular gyrus do not invariably possess the functions ascribed to them. As points of interest attention may be called to the localization of movements of the thumb and great toe. Occasionally they have found bilateral movements of a definite character follow stimulation of certain spots in one hemisphere—broadly put, the movements of the fingers, wrist, elbow, and shoulder as represented in the cortex of the ascending convolution in the order indicated, beginning from below. Certain definite movements have been observed in stimulation of the various parts of the internal capsule.

Dr. Angel Money's object has been to find out whether experimental irritation of the cerebral hemispheres has any power of causing movements in the plain muscular tissue of the stomach and intestines. The voluntary muscles were paralyzed by means of curare, thereby preventing muscular contraction on the application of the electrical currents to the surface of the cerebral hemispheres. The movements of the stomach and intestine were watched through a small window in the peritoneum. Artificial respiration was employed in the usual way. In some experiments the intestines were kept bathed in warm bath of neutral saline solution. The animals used were rabbits, dogs, and cats. In none of tnese animals did experimental electrical irritation of any part of the surface of the cerebral hemisphere cause movements of the stomach or intestines. When the cortex of the brain was removed by slicing, and electrical irritation practiced on the divided white matter, no result followed in the stomach or intestines unless a strong current was used. A movement of the stomach and intestines was invariably excited when the electrodes were applied to the basal region of the brain. This movement could be arrested by electrical irritation of the dorsal region of the spinal cord. It has been asserted by Vulpian that experimental irritation of the surface of the brain in the motor area can cause an epilepsy, so to speak, of the plain muscular tissue of the stomach and intestines. Dr. Money admits the difficulties of the inquiry, and has been unable to confirm this view. He believes that Vulpian's result may be attributed to diffusion of the current into the pneumogastic region.

Wm. B. Meany, M. D.

Glasgow, Aug. 11, 1888.

